# Lateral femoral condyle cartilage lesions in chronic posterior lateral meniscus root tears: A report of seven cases

**DOI:** 10.1002/jeo2.70194

**Published:** 2025-03-07

**Authors:** Alberto Grassi, Emanuele Altovino, Luca Ambrosini, Claudio Rossi, Luca Andriolo, Giuseppe Filardo, Stefano Zaffagnini

**Affiliations:** ^1^ Clinica Ortopedica e Traumatologica II, IRCCS Istituto Ortopedico Rizzoli Bologna Italy; ^2^ Department of Surgery, EOC Service of Orthopaedics and Traumatology Lugano Switzerland; ^3^ Faculty of Biomedical Sciences Università Della Svizzera Italiana Lugano Switzerland; ^4^ Applied and Translational Research (ATR) Center IRCCS Istituto Ortopedico Rizzoli Bologna Italy; ^5^ Dipartimento di Scienze Biomediche e Neuromotorie DIBINEM Università di Bologna Bologna Italy

**Keywords:** chronic, knee, meniscus, osteochondral lesion, root tears

## Abstract

This study aims to investigate the association between chronic lateral meniscus posterior root tears (LMPRTs) and chondral or osteochondral lesions of the lateral femoral condyle (LFC), particularly in cases involving prior anterior cruciate ligament (ACL) injuries. Given the challenges in diagnosing LMPRTs and the biomechanical significance of the lateral meniscus, this research highlights the potential long‐term impact of untreated root tears. A prospective analysis was conducted on seven patients with chronic LMPRTs and suspected LFC lesions, identified through clinical symptoms, history of ACL injuries and magnetic resonance imaging (MRI) findings. The presence of LMPRTs was confirmed via arthroscopy, and the lesions were classified using the LaPrade Classification. The patients underwent various surgical interventions, including ACL reconstruction with lateral tenodesis and meniscus repair. All seven cases demonstrated a significant association between chronic LMPRTs and chondral/osteochondral lesions of the LFC. The lesions were consistently located in the posterolateral compartment, with MRI indicating subchondral bone oedema and cartilage thinning. Surgical findings confirmed Type II posterior root tears in all patients, with subsequent repair. The study suggests that chronic LMPRTs, especially in the context of ACL injuries, may contribute to the development of chondral or osteochondral lesions in the LFC. This association underscores the importance of early diagnosis and treatment of meniscus root tears to prevent long‐term joint degeneration. Increased awareness and improved diagnostic techniques are essential for better clinical outcomes.

**Level of Evidence**: Level IV, case series.

**Abbreviations**
ACL: anterior cruciate ligament
LFC: lateral femoral condyle
LMPRT: lateral meniscus posterior root tear
MRI: magnetic resonance imaging
OA: osteoarthritis

## INTRODUCTION

Lateral meniscus root tears, particularly chronic posterior root tears (LMPRT), are significant yet often underdiagnosed pathologies in the knee joint with profound biomechanical and clinical implications. These injuries involve the detachment of the meniscus from its posterior attachment site on the tibia, disrupting its essential role in load distribution, shock absorption and joint stability [9, 19, 27].

The importance of these injuries is especially pronounced when they occur in conjunction with anterior cruciate ligament (ACL) injuries, which are frequently accompanied by meniscal damage [4, 13].

Biomechanically, the lateral meniscus functions as a critical component in maintaining knee joint stability and distributing mechanical loads across the joint surfaces [1]. Cadaveric studies have elucidated that the posterior root of the lateral meniscus acts as a secondary stabilizer, particularly in ACL‐deficient knees [12, 24]. The disruption of this stabilizing effect due to root tears results in increased joint laxity and altered load distribution, exposing the knee cartilage to possible damage and the development of osteochondral lesions. For instance, Shybut et al. demonstrated that root tears lead to increased joint pressure and decreased contact area, which could significantly contribute to the degeneration of articular cartilage [26]. This biomechanical insight highlights the essential role of the lateral meniscus root in supporting normal knee function.

Despite their clinical and biomechanical relevance, lateral meniscus root tears remain underreported, leading to potential long‐term joint issues [2]. In fact, their diagnosis poses significant challenges, primarily due to the limitations of current imaging modalities. Magnetic resonance imaging (MRI) is the standard diagnostic tool, yet its sensitivity and specificity for detecting root tears are suboptimal. Many authors observed that MRI demonstrates the lowest sensitivity for detecting tears located in the posterior horn of the lateral meniscus, especially when compared to tears in other meniscal regions [7]; the reduced sensitivity is particularly pronounced in patients with concomitant ACL injuries [11].

Indirect signs on MRI, such as the ‘ghost sign’ (absence of the normal meniscal signal) and lateral meniscal extrusion on coronal view, can suggest the presence of a root tear, but these signs are not always present or easily identifiable [9]. Consequently, there is a risk of delayed or missed diagnosis, which can lead to untreated tears [16]. If the detrimental biomechanical effects of lateral meniscus root tears have been broadly demonstrated in a multitude of cadaveric models [8, 30], the long‐term clinical consequences have not been investigated in depth, and high‐level evidence is still lacking. The most important study on this topic has been performed by Shelbourne et al., who compared the outcomes of 33 patients with untreated lateral root tears and ACL reconstruction to a matched group of patients with isolated ACL reconstruction and intact menisci. Despite no clinically meaningful differences were reported at a minimum follow‐up of 5 years, a significantly higher joint space narrowing was reported in patients with untreated root tears [25].

In light of these considerations, the present study aims to document a series of patients with a peculiar combination of lateral femoral condyle (LFC) cartilage lesions and chronic LMPRT. The objective is to contribute to the growing body of evidence regarding the impact of these injuries and to highlight the necessity for improved diagnostic and therapeutic strategies.

## METHODS

As this was a prospective study involving normal clinical practice and standard treatment, informed consent was not necessary. Seven cases of chondral and osteochondral lesions of the LFC in patients with suspected chronic LMPRT were prospectively identified by an orthopaedic surgeon (A.G.) in one single Institution (Istituto Ortopedico Rizzoli, University of Bologna, Italy) based on medical history, symptoms and imaging between January 2023 and December 2024.

The diagnosis of chondral/osteochondral lesion of the LFC was based on lateral knee pain and hyperintense signal in T2‐weighted MRI on the posterolateral femoral condyle, associated with cartilage thinning and abnormal signal. Clinical suspicion of the presence of an LMPRT was based on the history of previous ACL injury or isolated ACL reconstruction without meniscal treatments. Moreover, indirect signs of root tears in knee MRI, such as the ‘ghost sign’ and meniscal extrusion, were investigated to confirm the suspect of the lesion. In all patients, the presence of the LMPRT was confirmed during arthroscopy at the time of treatment and classified according to the LaPrade Classification [3, 17].

## RESULTS

### Case 1

A 29‐year‐old male recreational basketball player sustained a non‐contact left knee sprain 4 years before with the diagnosis of an isolated ACL tear. Due to the absence of subjective instability, the patient was treated conservatively in another institution. Two years after the initial trauma, he started to complain of lateral knee pain and recurrent swelling, however, without any traumatic event.

**Figure 1:**
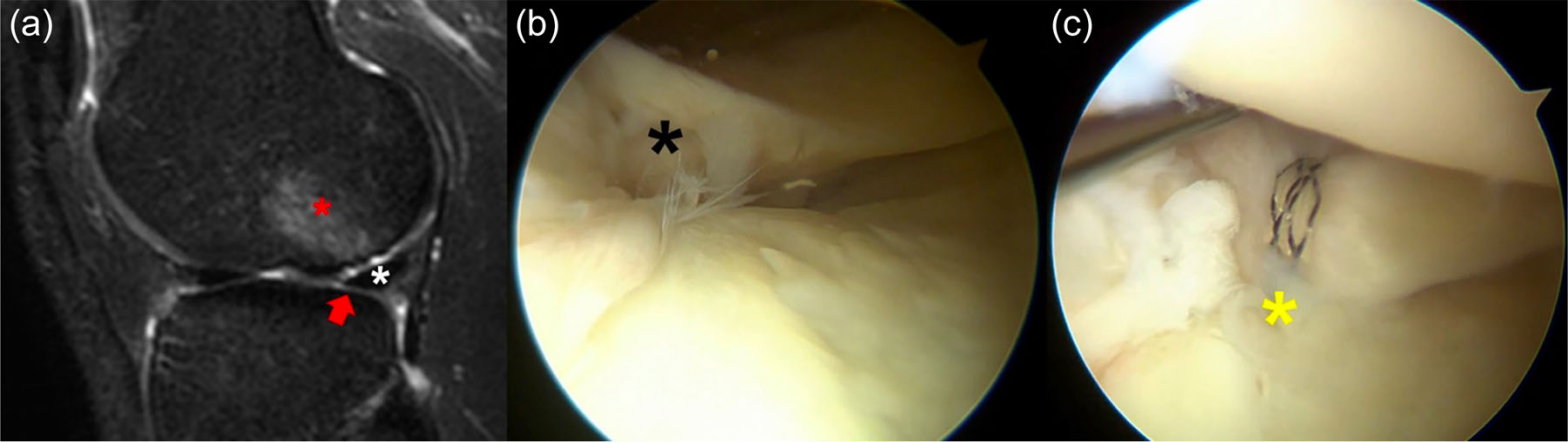
Left knee of a 29‐year‐old patient with a chronic ACL tear. The sagittal MRI of the lateral compartment shows the presence of subchondral bone oedema (red asterisk) and cartilage thinning (red arrow) at the lateral femoral condyle, with an intact lateral meniscus (white asterisk) (a). At the time of arthroscopy, the posterior root (black asterisk) was torn from the tibial insertion and floating (b). The root (yellow asterisk) was repaired with a transtibial tunnel (c). ACL, anterior cruciate ligament; MRI, magnetic resonance imaging.

At the time of presentation, the patient reported lateral knee pain and recurrent swelling during mild physical activity. He had Lachman 1+ ,Pivot‐Shift 2+ and Anterior Drawer 1+ . MRI showed an area of hyperintense signal in the non‐weight‐bearing area of the posterolateral femoral condyle in sagittal T2 slices, with a focal area of cartilage loss (Figure 1a). The posterolateral root was not clearly visible, although no ‘ghost sign’ was noted. Due to the clinical and imaging presentation, a concomitant chronic root tear was suspected.

At the time of arthroscopy for ACL reconstruction, lateral tenodesis, and autologous minced cartilage resurfacing, a complete Type II posterior root tear was noted (Figure 1b), which was repaired with a pullout trans‐tibial suture (Figure 1c).

### Case 2

A 38‐year‐old male tennis coach sustained a non‐contact left knee sprain 15 years before at the age of 23, with the diagnosis of isolated ACL tear. Due to the absence of subjective instability, the patient was treated conservatively in another institution. Since the patient remained active without ACL reconstruction, he underwent a medial meniscectomy due to a secondary bucket handle tear of the medial meniscus 7 years later. In the last 2 years, he started to complain of lateral knee pain and recurrent swelling, however, without any traumatic event.

**Figure 2:**
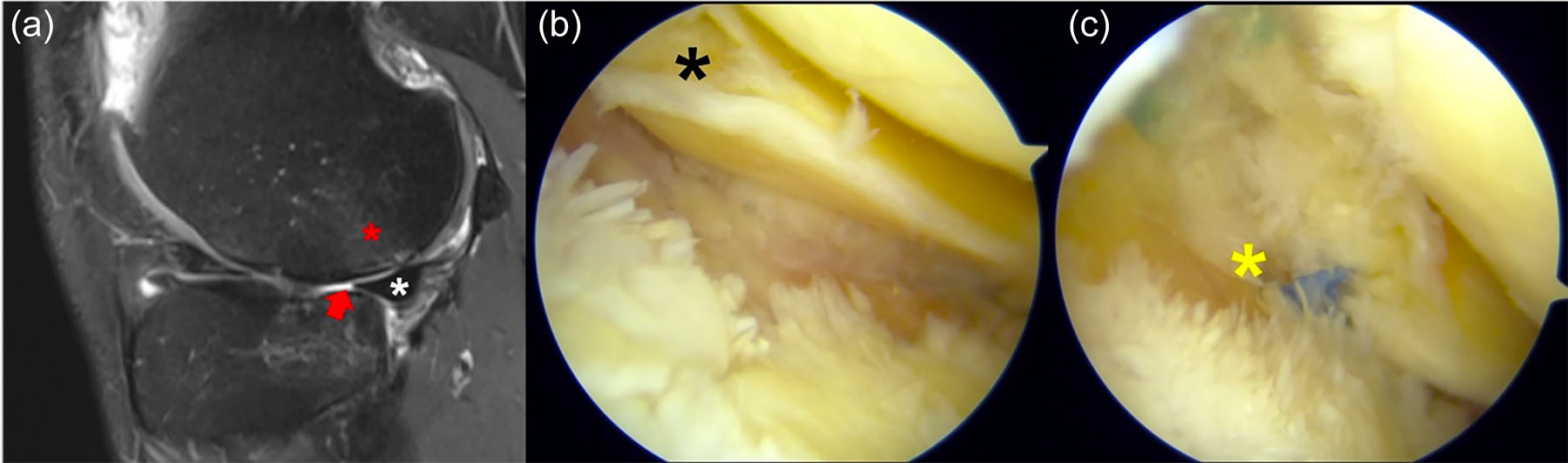
Left knee of a 38‐year‐old patient with a chronic ACL tear. The sagittal MRI of the lateral compartment shows the presence of subchondral bone oedema (red asterisk) and cartilage thinning (red arrow) at the lateral femoral condyle, with an intact lateral meniscus (white asterisk) (a). At the time of arthroscopy, the posterior root (black asterisk) was torn from the tibial insertion and floating (b). The root (yellow asterisk) was repaired with a transtibial tunnel (c). ACL, anterior cruciate ligament; MRI, magnetic resonance imaging.

At the time of presentation, the patient complained of lateral knee pain and recurrent swelling during mild physical activity. He had Lachman 2+ , Pivot‐Shift 3+ and Anterior Drawer 2+ . MRI showed an area of hyperintense signal in the non‐weight‐bearing area of the posterolateral femoral condyle in sagittal T2 slices, with a focal area of cartilage loss both in the femoral condyle and tibial plateau (Figure 2a). The posterolateral root was not clearly visible, although no ‘ghost sign’ was noted. Despite the previous meniscectomy in the medial compartment, signs of joint degeneration were noted in the lateral compartment. Due to the clinical and imaging presentation, a concomitant chronic root tear was suspected. At the time of arthroscopy for ACL reconstruction plus lateral tenodesis, a complete Type II posterior root tear was noted (Figure 2b), which was repaired with a pullout trans‐tibial suture (Figure 2c).

### Case 3

**Figure 3:**
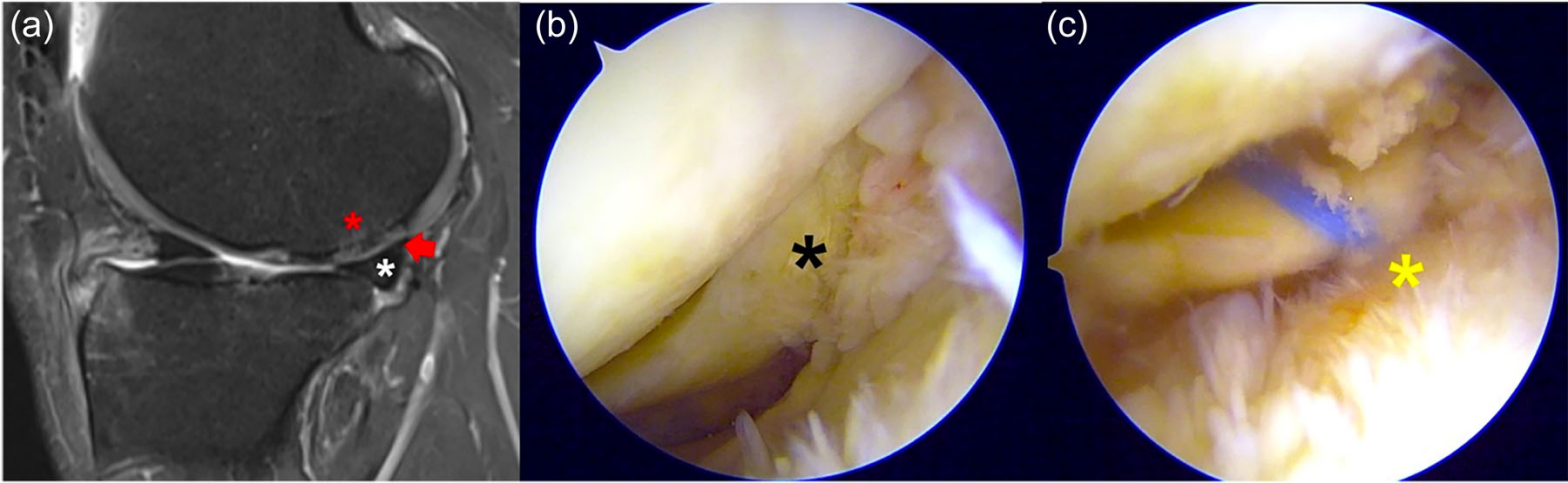
Right knee of a 39‐year‐old patient with chronic ACL tear. The sagittal MRI of the lateral compartment shows the presence of subchondral bone oedema (red asterisk) and cartilage thinning with tissue inhomogeneity and abnormal signal (red arrow) at the lateral femoral condyle, with an intact lateral meniscus (white asterisk) (a). At the time of arthroscopy, the posterior root (black asterisk) was torn from the tibial insertion and floating (b). The root (yellow asterisk) was repaired with a transtibial tunnel (c). ACL, anterior cruciate ligament; MRI, magnetic resonance imaging.

The same 38‐year‐old patient (Case 2) underwent the same procedure in the contralateral right knee, where he had a non‐contact ACL injury 13 years before and a medial meniscectomy 6 years before, with a similar presentation as the left knee (Figure 3a). At the time of arthroscopy for ACL reconstruction plus lateral tenodesis, a complete Type II posterior root tear was noted (Figure 3b), which was repaired with a pullout trans‐tibial suture (Figure 3c).

### Case 4

An 18‐year‐old male recreational footballer sustained a non‐contact right knee sprain 2 years before, at the age of 16, with the diagnosis of an isolated ACL tear. Due to skeletal immaturity and the absence of subjective instability, the patient was treated conservatively in another institution. After 1 year from the initial trauma, he started to report lateral knee pain and recurrent swelling, however, without any new traumatic event.

**Figure 4:**
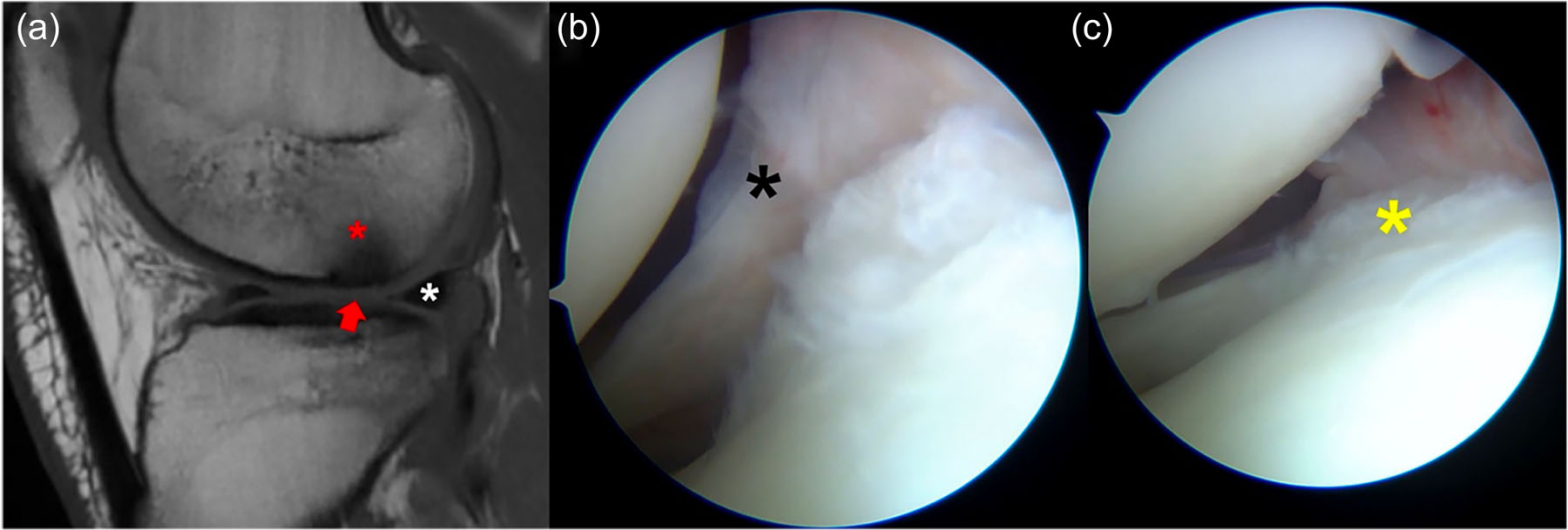
Right knee of an 18‐year‐old patient with a chronic ACL tear. The sagittal MRI of the lateral compartment shows the presence of subchondral bone oedema (red asterisk) and cartilage thinning (red arrow) at the lateral femoral condyle, with an intact lateral meniscus (white asterisk) (a). At the time of arthroscopy, the posterior root (black asterisk) was torn from the tibial insertion and floating (b). The root (yellow asterisk) was repaired with an all‐inside suture of the remnant (c). ACL, anterior cruciate ligament; MRI, magnetic resonance imaging.

At the time of presentation, the patient complained of lateral knee pain and recurrent swelling during mild physical activity. The clinical exam showed Lachman 3+, Pivot‐Shift 3+ and Anterior Drawer 3+. MRI showed an area of altered signal in the non‐weight‐bearing area of the posterolateral femoral condyle in sagittal T1 slices, with a focal area of cartilage thinning (Figure 4a). The posterolateral root was not clearly visible, although no ‘ghost sign’ was noted. Due to the clinical and imaging presentation, a concomitant chronic root tear was suspected.

At the time of arthroscopy for ACL reconstruction and lateral tenodesis, a complete Type II posterior root tear was noted (Figure 4b), which was repaired with an all‐inside suture of the remnant (Figure 4c).

### Case 5

A 33‐year‐old male recreational athlete underwent isolated ACL reconstruction 8 years before. At the time of surgery, no meniscal lesions were reported. In the last 3 years, the patient started to complain of lateral knee pain despite the treatment with hyaluronic acid injections.

**Figure 5:**
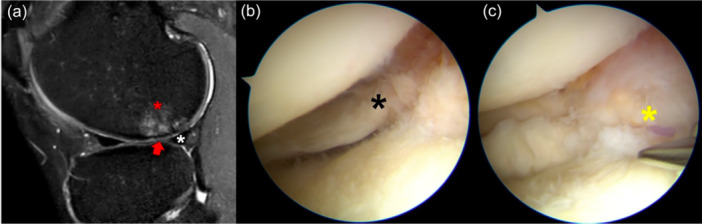
Right knee of a 33‐year‐old patient with previous isolated ACL reconstruction. The sagittal MRI of the lateral compartment shows the presence of subchondral bone oedema (red asterisk) and cartilage thinning, inhomogeneity, and abnormal signal (red arrow) at the lateral femoral condyle, with an intact lateral meniscus (white asterisk) (a). At the time of arthroscopy, the posterior root (black asterisk) was torn from the tibial insertion and floating (b). The root (yellow asterisk) was repaired with an all‐inside suture of the remnant (c). ACL, anterior cruciate ligament; MRI, magnetic resonance imaging.

At the time of presentation, the patient reported lateral knee pain and recurrent swelling during mild physical activity. He had negative Lachman, Pivot‐Shift and Anterior Drawer. MRI showed a continuous ACL graft, with a hypointense signal and an area of hyperintense signal in the non‐weigh‐bearing area of the posterolateral femoral condyle in sagittal T2 slices, with a focal area of cartilage thinning, inhomogeneity and abnormal signal (Figure 5a). The posterolateral root was not clearly visible, although no ‘ghost sign’ was noted. Due to the clinical and imaging presentation, a concomitant chronic posterior root tear of the lateral meniscus was suspected.

At the time of arthroscopy for autologous minced cartilage resurfacing, a complete Type II posterior root tear was noted (Figure 5b), which was repaired with an all‐inside suture of the remnant (Figure 5c).

### Case 6

**Figure 6:**
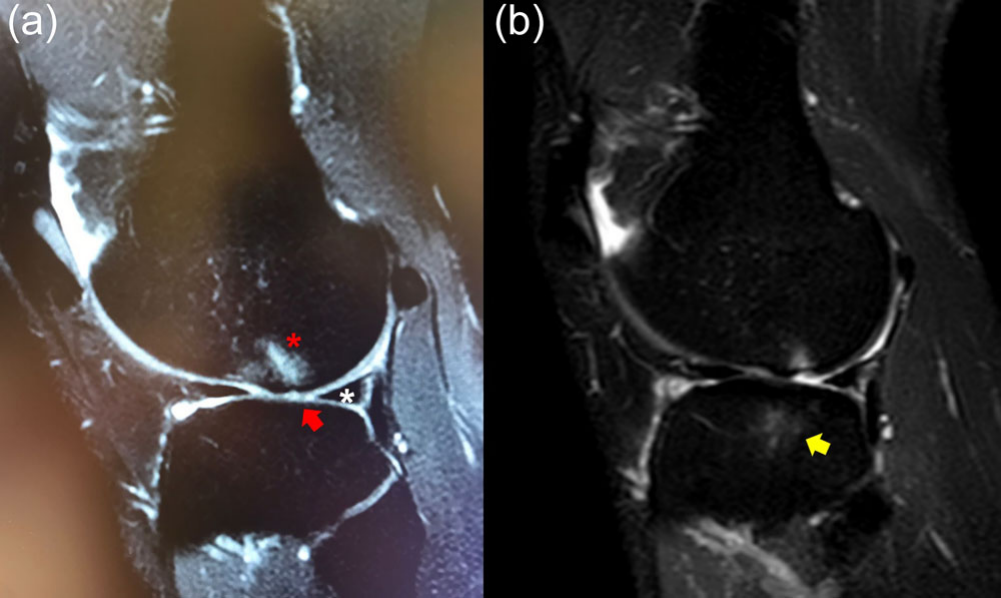
Left knee of a 27‐year‐old patient with a chronic ACL tear. The sagittal MRI of the lateral compartment shows the presence of subchondral bone oedema (red asterisk) and cartilage thinning (red arrow) at the lateral femoral condyle, with an intact lateral meniscus (white asterisk) (a). After 4 years, at the age of 31, the cartilage defect of the lateral femoral condyle was more evident, and bone oedema in the lateral tibial plateau was present as well (yellow arrow) (b). ACL, anterior cruciate ligament; MRI, magnetic resonance imaging.

A 31‐year‐old male competitive footballer player sustained a non‐contact left knee sprain 4 years before at the age of 27, with the diagnosis of isolated partial ACL tear. Due to the absence of subjective instability, the patient was treated conservatively in another institution. After 1 year from the initial trauma, he started to complain of lateral knee pain and mild instability. He underwent a knee MRI, which showed bone oedema of the LFC (Figure 6a). With conservative treatment, the player was able to return to sport. However, after another 2 years, pain and instability increased without any new relevant traumatic event.

At the time of presentation, after 1 year of conservative treatment, the patient still reported lateral knee pain during football practice. He had Lachman 1+, Pivot‐Shift 2+ and Anterior Drawer 1+. MRI showed an area of hyperintense signal in the non‐weight‐bearing area of the posterolateral femoral condyle and tibial plateau in sagittal T2 slices, with a focal area of cartilage loss (Figure 6b). The posterolateral root was not clearly visible, although no ‘ghost sign’ was noted. Due to the clinical and imaging presentation, a concomitant chronic root tear was suspected.

At the time of arthroscopy for ACL reconstruction and lateral tenodesis, a complete Type II posterior root tear was noted, which was repaired with an all‐inside suture of the remnant.

### Case 7

**Figure 7:**
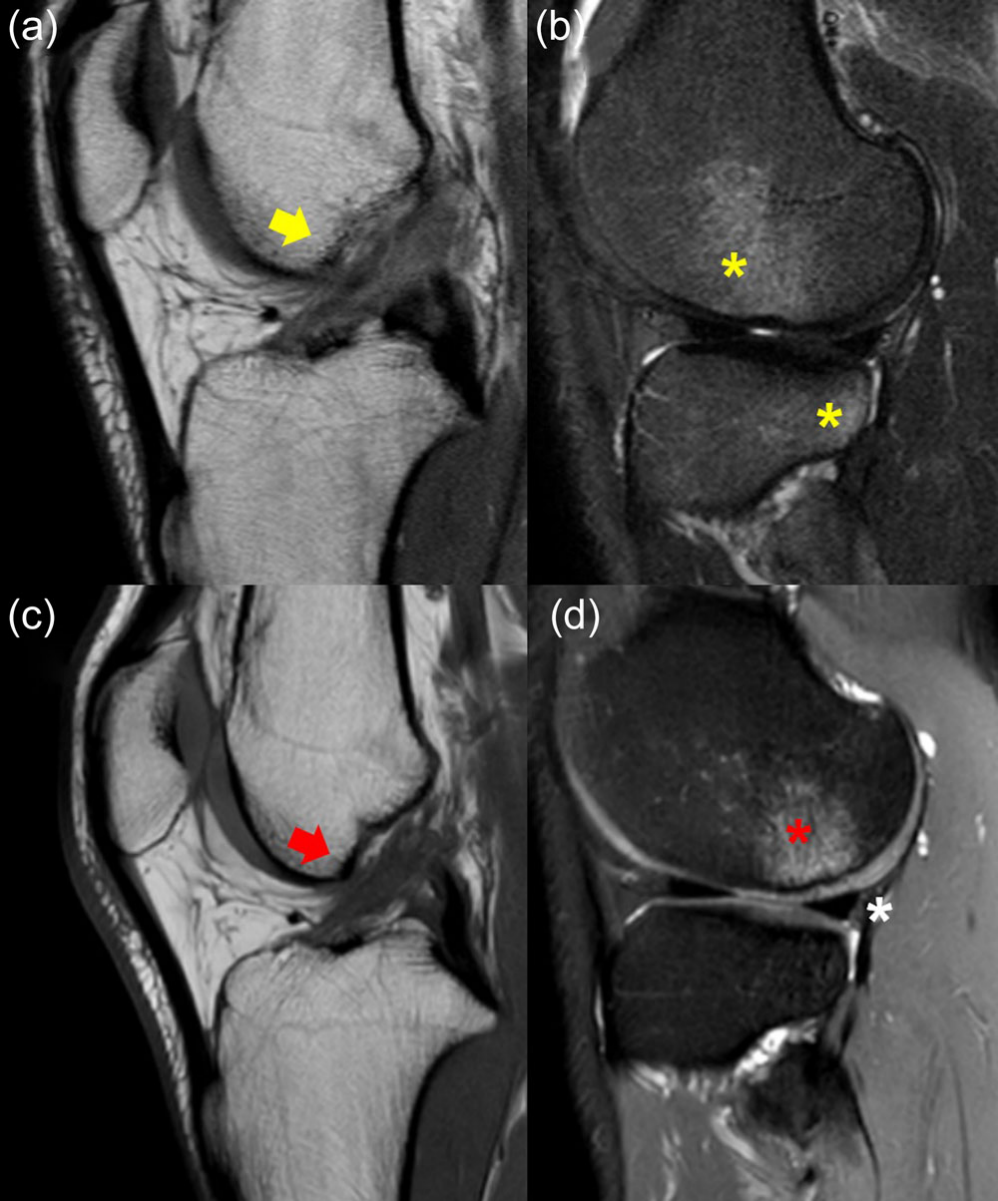
Left knee of a 22‐year‐old patient with an acute partial ACL tear. The sagittal MRI showed altered signal and continuity of the ACL (yellow arrow) (a) and the classic ACL‐injury‐related bone bruises of the anterior lateral femoral condyle and posterolateral tibial plateau (yellow asterisks) (b). After 4 years, at the age of 26, the sagittal MRI shows the altered signal of the ACL (red arrow) (c) and the presence of subchondral bone oedema (red asterisk) in the posterior portion of the lateral femoral condyle, with an intact lateral meniscus (white asterisk) (d). ACL, anterior cruciate ligament; MRI, magnetic resonance imaging.

A 26‐year‐old male competitive footballer player sustained a non‐contact left knee sprain 4 years before at the age of 22, with the diagnosis of isolated partial ACL tear and classic tibio‐femoral bone bruises (Figure 7a,b). Due to the absence of subjective instability, the patient was treated conservatively in another institution. 3 years after the initial trauma, he started to complain of lateral knee pain during change of direction.

At the time of presentation, after 1 year of failed conservative treatment, the patient still reported lateral knee pain during football practice. He had Lachman 1+, Pivot‐Shift 2+ and negative Anterior Drawer. MRI showed an area of hyperintense signal in the non‐weight‐bearing area of the posterolateral femoral condyle and tibial plateau in sagittal T2 slices (Figure 7c,d). The posterolateral root was not clearly visible, although no ‘ghost sign’ was noted. Due to the clinical and imaging presentation, a concomitant chronic root tear was suspected.

At the time of arthroscopy for ACL reconstruction, lateral tenodesis, and autologous minced cartilage resurfacing, a complete Type II posterior root tear was noted, which was repaired with an all‐inside suture to the remnant.

## DISCUSSION

The main finding of this study is that chronic LMPRT may be associated with lesions of the articular surface, especially in the posterior portion of the LFC.

LMPRTs are known to be a frequent finding combined with ACL injuries, a relationship that was confirmed in every patient presented in this series. Historically, ACL injuries have been considered to be frequently associated with subchondral alterations, cartilage lesions, and degenerative joint evolution, such as osteoarthritis (OA) [18, 23]. Despite evidence showing a strong aetiological association between ACL tears and the subsequent development of knee OA, it is not clear if the accelerated degeneration of cartilage leading to OA is caused by the mechanical instability and altered joint biomechanics resulting from an ACL tear or from the detrimental effect of the associated meniscal tears. In fact, the loss of meniscal function due to lesions or defects is a well‐established risk factor for knee OA [10, 15, 32]. Considering the difficult diagnosis of meniscal tears such as the root and ramp ones, it could be speculated that such lesions could pass unnoticed in the case of ACL tear more than we think, and this raises the possibility that their detrimental effect on knee biomechanics may play a more significant role in the subsequent degeneration of the knee joint rather than the ACL injury itself.

**Figure 8:**
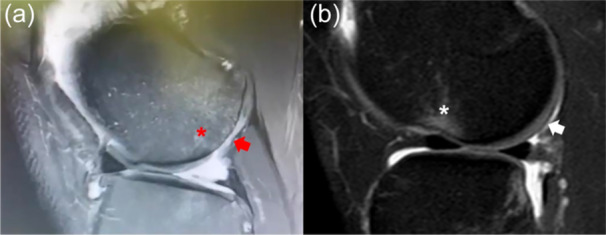
Sagittal MRI showing the subchondral bone oedema (red asterisk) and cartilage loss (red arrow) in the posterior area of the LFC in a 26‐year‐old patient who sustained subtotal lateral meniscectomy 8 years before (a). Sagittal MRI of an acute ACL tear, with signs of traumatic subchondral bone oedema in the anterior portion of the LFC (white asterisk) and intact cartilage in the posterior part (white arrow) (b). ACL, anterior cruciate ligament; LFC, lateral femoral condyle; MRI, magnetic resonance imaging.

In this regard, while most of the historical studies [6, 28, 29] demonstrated the role of ACL injury in the development of knee OA, the specific role of LMPRT is less investigated. Some findings lead to the hypothesis that the articular surface lesions reported in the current series may not be related to the altered kinematics related to the ACL injury, but to the meniscal tear itself. In fact, chondral wear and articular lesions related to ACL injury are more frequently detected in the medial compartment than in the lateral compartment, which was affected instead in the patients analyzed in this series [31]. Further supporting the role of the meniscal lesion in determining the cartilage damage, it is worthy of note that the location of the damage in this series highly resembles the one derived from partial meniscectomy of lateral meniscus posterior horn lesion (Figure 8a), and that it does not coincide with the location of the bone bruise related to ACL rupture, which is found in the anterior part of the LFC (Figure 8b). Consequently, considering the inherent difficulty of root tear diagnosis by MRI, the posterior bone bruise in a knee without a previous meniscectomy may potentially be used as a secondary diagnostic sign of chronic LMPRT. However, further studies should analyze deeper and confirm this association.

The explanation of these tears may be found biomechanically in the loss of meniscal hoop function related to the LMPRT and in the altered function of secondary stabilization. In fact, the posterior root of the lateral meniscus also plays an important role as a secondary restraint against the pivot shift in ACL‐deficient knees, as demonstrated by its increase in ACL‐deficient knees after root tears in a cadaveric study [26].

Clinically, it has also been shown that LMPRTs are associated with a larger pivot shift grade in ACL‐injured knees [20, 21]. This pivot shift may explain the location of the cartilage damage in this small series. Considering the high lateral meniscus mobility, it is possible that the lack of connection between the tibial plateau and the posterior meniscal horn caused a dynamic and non‐physiological meniscus displacement during knee flexion and rotation, thus leading to posterolateral cartilage overload and wearing.

The clinical and radiological long‐term consequences of chronic LMPRT remain to be clarified. In the most important study about this subject, Shelbourne et al. concluded that at a mean of 10 years follow‐up, LMPRTs left in situ were associated with mild lateral joint‐space narrowing without significant differences in subjective or objective scores compared with controls [25]. Nevertheless, as suggested by the patients of the present case series, chondral and osteochondral lesions may also occur in a few years; thus, an early diagnosis should be encouraged. LMPRTs should be investigated and suspected at the first visit after an ACL injury according to direct and indirect MRI signs, confirmed during arthroscopy, and treated properly to improve the stability of the joint, as demonstrated by several cadaveric studies [14, 22]. Moreover, it cannot be excluded that root tears, and meniscal tears in general, may occur in a second moment in ACL deficient knees, and this may suggest being careful in treating conservatively ACL ruptures [5], especially in active patients where ACL reconstruction may be suggested even in the case of limited instability when they have a suspected or confirmed LMPRT.

This study presents several limitations. In the first instance, it is only a report of seven cases, with heterogeneous characteristics regarding the timing of the lesion, the different clinical presentations, and the radiological findings. The only common finding in this small report is the association of chronic LMPRT with a chondral/osteochondral lesion of the LFC. Moreover, it has to be noted that six cases were associated with untreated ACL tears at the time of the first clinical examination. Consequently, no definitive evidence can be proposed regarding the etiopathological role of the meniscus compared to ACL or other anatomical and biomechanical aspects not considered in this case report. Larger case‐control studies with patients with untreated root tears and reconstructed ACL and untreated ACL without root tears should further analyze this relationship. Moreover, the role of the treatment of these meniscal lesions in the natural history of the lateral compartment should be analyzed. Nevertheless, this series underlines a potentially important association between LMPRTs and chondral/osteochondral lesions of the LFC.

## CONCLUSIONS

In the present report of seven patients, a chronic LMPRT after ACL injury was associated with a posterolateral chondral or osteochondral lesion of the LFC. This association could also be used as a secondary criterion for root tear diagnosis. This initial evidence supports the need to increase awareness of the clinical consequences of untreated meniscal root tears.

## AUTHOR CONTRIBUTIONS

All authors contributed to the study conception and design. Material preparation, data collection and analysis were performed by Alberto Grassi, Claudio Rossi and Luca Ambrosini. The first draft of the manuscript was written by Alberto Grassi, Luca Andriolo, Emanuele Altovino, Giuseppe Filardo and Stefano Zaffagnini. All authors commented on previous versions of the manuscript. All authors read and approved the final manuscript.

## CONFLICT OF INTEREST STATEMENT

Stefano Zaffagnini is consultant for De Puy Synthes and Smith&Nephew and Editor in Chief of JEO journal. The remaining authors declare no conflicts of interest.

## ETHICS STATEMENT

The ethics statement is not available.

## Data Availability

Data sharing not applicable to this article as no data sets were generated or analysed during the current study.
